# Expert communication on Twitter: Comparing economists’ and scientists’ social networks, topics and communicative styles

**DOI:** 10.1177/0963662520957252

**Published:** 2020-09-15

**Authors:** Marina Della Giusta, Sylvia Jaworska, Danica Vukadinović Greetham

**Affiliations:** University of Reading, UK; The Open University, UK

**Keywords:** communicative style, expert communication, involvement, networks, sentiment, Twitter

## Abstract

Experts increasingly use social media to communicate with the wider public, prompted by the need to demonstrate impact and public engagement. While previous research on the use of social media by experts focused on single topics and performed sentiment analysis, we propose to extend the scope by investigating experts’ networks, topics and communicative styles. We perform social and semantic network as well language analysis of top tweeting scientists and economists. We find that economists tweet less, mention fewer people and have fewer Twitter conversations with members of the public than scientists. Scientists use a more informal and involved style and engage wider audiences through multimedia contents, while economists use more jargon, and tend to favour traditional written media. The results point to differences in experts’ communicative practices online, and we propose that disciplinary ways of ‘talking’ may pose obstacles to an effective public communication of expert knowledge.

## 1. Introduction

Communication of expert knowledge to a lay audience has been high on the agenda since the end of World War II (Stilgoe et al., 2014). Since then, we have witnessed seven decades of exponential growth in science and technology fundamentally changing our lives at a very fast pace. Never before have science, technology and other branches of knowledge played such a significant role in everyday life, and there has never been a greater interest in scientific matters by lay audiences. Yet, the relationship between experts and the wider society is often far from cordial, and the ways in which experts communicate tend to cause more distrust and confusion rather than trust and understanding. The rejection of expertise, which in the context of the United Kingdom culminated in the public outcry by the former UK education secretary Michael Gove (2016) that the British people ‘have had enough of experts’ is a telling reflection of this wider public mistrust towards experts and expertise. This attitude has only partially to do with the complexity of the issues with which experts engage. It is also due to the perception that experts have of lay people. For some time, the understanding held sway that a lay person operates within a cognitive deficit and does not know enough about, for example, science or finance to comprehend what experts do (Irwin and Wynne, 1996; Poon and Olen, 2015). It is then the task of the expert to tell the lay people what they need to know and how they ought to think and behave. Some of the communication fiascos of the 1990s including the BSE crisis, GM food and vaccinations have shown the inefficiency and sometimes disastrous consequences of this one-way and top-down model of communication.

Since then, a new model of science communication has been encouraged based on a two-way engagement with the public. Digital technologies, but especially social media sites have opened up a whole host of new opportunities for engagement with lay people with some enthusiasts heralding them as new important tools of popularising and democratising knowledge. Although the enthusiasm for the role of social media in democratic processes has been dampened by recent data scandals, the up-take of social media sites by experts has been considerable. Scientists in particular seem to have realised quite quickly the potential of social media for engaging the public and have been utilising various social media platforms to support research and research communications (Darling et al., 2013; Lupton, 2014; Rowlands et al., 2011; Scheufele, 2014; Shiffman, 2012; Van Noorden, 2014; Walter et al., 2018, 2019). Compared to other groups of experts, scientists seem to be ‘the biggest users of social media’ (Rowlands et al., 2011: 186). Other experts including economists too began using social media sites to communicate about their subject matters; yet given the current distrust towards economists and economic expertise, it seems that their social media communications have not necessarily contributed to a better engagement with the public. This may have to do as much with what they talk about as with how they do it.

It is precisely the ‘how’ of social media communication that this study seek to explore. Much of previous research on the use of social media platforms by experts was primarily concerned with topics and with whom experts communicate on these topics (networks). Most attention was dedicated to scientists, which is not surprising, given that they utilise social media platforms quite extensively. In terms of the topics, most studies to date have focused on climate change (e.g. Jahnng and Lee, 2018; Walter et al., 2019). While retrieving topics that experts talk about on social media gives us some understanding of what kind of themes are deemed to be significant and broadcast to the wider public, it still does not tell us much about how the matters are communicated. Studying the ‘how’ of communication is relevant for at least two reasons; first, complex or abstract subject matters can always be explained in a language which can be easier understood by members of the public. Generally, less complexity and more informality can assist understanding, whereas more formal style based on expert jargon can be difficult or even impossible to ‘digest’ by non-experts leading to resistance in engaging with the information (Bullock et al., 2019). Studying the degree of formality versus informality in social media communications by experts can shed light on how expert knowledge is communicated to the wider public. Second, exploring the strategies of how experts communicate on social media sites, specifically those who engage the wider public, could help us identify some of the successful strategies of knowledge dissemination and engagement.

This study investigates the communicative practices of the 25 most followed scientists and compares them to that of the 25 most followed economists, who together account for the vast majority of the following (compared to others listed as lower ranking), specifically 25,681,691 followers for scientists and 7,063,217 followers for economists. The choice of the 25 most followed scientists and economists ensures a focus on the group of experts who have a wider reach and resonance within the Twitter communities as well as the disciplines with the greatest influence on public policy (Côté and Darling, 2018; Coyle, 2012; Fourcade et al., 2015). In contrast to previous research, which mostly focused on a single topic or a network analysis, this study is much more comprehensive in scope in that it investigates three dimensions of expert communication: networks, topics and style.

Following the sociolinguistic conceptualisation of communicative style (Coupland, 2007), we understand the notion as a *way* of talking and a communicative repertoire comprising distinctive linguistic (lexical and grammatical) features and paralinguistic resources (e.g. articulation). These features, all put together, can indicate the level of formality (non-involved style) versus informality (involved style). Essentially, the formal style, which is by many regarded the typical dense style of written expert communication, includes references to specialist vocabulary (expert jargon), nominalisations (condensing information in nouns and noun phrases), complex words, passive and long sentences with subordinate clauses and fewer or no emotive lexis (Biber and Conrad, 2009). In contrast, the involved style is typical of informal conversations and consists of features such as the use of personal pronouns, discourse markers (*yeah, like, you know*), intensifiers (*extremely, absolutely*), downtowners (*pretty, kind of*), emotive and evaluative lexis (*crazy, terrible*) as well as swear and slang words (Biber and Conrad, 2009).

Initially, Twitter was conceived as a microblogging platform for information exchange and the early writing on Twitter exhibited a journalistic and more formal style. Yet, the subsequent inclusion of new interactional features such replies, the like button, mentions and as well as the opportunity to use multimodal resources (emojis, videos, etc) have encouraged a more conversational and informal style of communication. We are interested in the extent to which the two groups of experts utilise these affordances and whether there are any differences and similarities between the scientists and economists in terms of the communicative style, specifically at the level of formality and involvement. To do so, we explore features of formality and informality in the top 100 keywords and key terms used by both groups of experts. Keywords and key terms are words and combinations of words that are distinctive of and routinely used in a given set of textual data as compared to a larger reference corpus. They are considered useful indicators of topics and style (e.g. Baker, 2017; Charteris-Black, 2012). Studying keywords and key terms allows us therefore to reveal not only what the most followed scientists and economists communicate about, but more importantly *how* they do it.

Because the notion of style depends very much on the context of situation and who communicates with whom and what about, we also undertake a network and semantic analysis to understand who the most followed scientists and economists engage with and what they communicate about on Twitter. This is supported by the analysis of mentions of people, which are regarded as conversational tools and useful indicators of engagement and collaboration (Moulaison and Burns, 2012). Research has shown that tweets with mentions are more likely to receive public responses (Honeycutt and Herring, 2009). Investigating the number of mentions and who is mentioned by the two groups of experts can therefore reveal with whom scientists and economists engage on Twitter and the extent of their reach to the public.

## 2. Expert communication on Twitter

Ever since the late 1990s, scientists have begun participating in more interactive forms of communication with the view to engage the public. Interactive science programmes began to appear on public and private broadcasting channels (examples from the BBC include *People of Science* or *Wonders of the Universe*) and prominent scientists have been regular speakers on TED talks since its inception (Davies and Horst, 2016). Initiatives such as citizen science have been launched to increase the participation of non-scientists in science and to produce scientific research which responds to the needs and concerns of the public (Bonney et al., 2014). Possibly because of these developments, scientists fairly quickly realised the potential of social media platforms to communicate with peers and others. While for most experts, specifically academic experts, media and social media are still ‘not a natural habitat’ (Tirole, 2017: 72), scientists use social media actively, Twitter in particular, to communicate with other scientists and members of the public (López-Goñi and Sánchez-Angulo, 2018; Walter et al., 2019).

Twitter is a microblogging social media platform where users can post short texts (up to 280 characters in length) for viewing by other users. Although initially conceived as a tool of information exchange, Twitter became quickly a *conversational* platform afforded by technical devices such as replies, retweets, the like button, hashtags and mentions, all of each foster interactivity, engagement and the creation of affiliations in the Twittersphere (e.g. boyd, 2011; Zappavigna, 2014). Both the information exchange and the interactional aspects have made Twitter the most popular social media tool used by experts (Lupton, 2014). They value Twitter because it helps them keep abreast with the developments in their fields (e.g. conference tweeting); those who work within academic institutions see it as excellent source of information about grant opportunities and new policies, while the connectivity with peers offers opportunities for finding collaborators, brainstorming ideas and receiving feedback (e.g. Bonetta, 2009). Research shows that Twitter can lead to some quantifiable academic ‘returns’ in that it can speed up the life cycle of publications. There is some evidence to suggest that papers which are tweeted receive more citations that non-tweeted ones (Peoples et al., 2016).

While in-reach is the key activity of experts who tweet, they do not only communicate with peers. For example, Côté and Darling (2018) have shown that 40% of the followers of scientists with a large follow-ship (more than ∼1000) were representatives of the public, media, applied organisations, outreach groups and decision makers. The authors concluded that as the number of followers increase, so does the number of users who represent non-academic audiences. Thus, having more followers increases the diversity of audiences and reach of scientific messages. This also suggests that tweeting practices of the most followed experts can be regarded, in many ways, as examples of wider outreach.

Exploring tweeting practices of scientists on the topic of climate change, Walter et al. (2019) found that scientists develop a distinctive style of communication on Twitter, which is different from ‘pure’ scientific talk. They also adjust their language to different audiences. This suggests that scientists exploit the medium and its affordances to foster a more strategic communication, which, the authors argue, can lead to a better engagement with scientific knowledge outside the scientific community and a better public understanding of science. Yet, the analysis of language in this study is based only on a sentiment analysis. While sentiment analysis can offer some general insights into the positivity or negativity of a message (as we show below), it does not reveal any other dimensions of language use and tells us little about the communicative style of expert communication.

Turning to economics and economists, research concerned with the communicative style of economists suggest that they display a higher degree of assertiveness, formality and a sense of superiority, which are linked to the higher remunerations, insularity and hierarchy that exist in the discipline relative to the other branches of sciences and social sciences (Fourcade et al., 2015). Some argue that this has to do with the unrealistic model of human beings as cold calculating machines which largely prevails the thinking in the discipline, contributing to its inability to produce realistic and useful models, and has failed to produce appropriate public communication models (Bowles and Gintis, 1993; Nelson, 1995). More recently, arguments about arrogance have featured in parallel discussions about the way in which economists and the discipline itself are perceived by the public and by potential undergraduate recruits (Tonin and Wahba, 2015). Yet, little is known about the communicative practices of economists. Being a public archive of utterances by leading economists accompanied by measures of their resonance, Twitter offers a unique opportunity to test these practices and investigate the ways in which they engage with the public in this medium.

Assuming that experts with large follow-ships on Twitter can be regarded as engaged expert communicators, we investigate in more detail networks, topics and communicative style of the top 25 most followed scientists and economists. We explore in particular differences and similarities in terms of their audiences (*with whom* they communicate), *what* they communicate *about* and *how* they do it. The specific research questions that this study addresses are:

1. With whom do the most followed 25 scientists and economists communicate?
2. What do they communicate about?
3. Is there evidence to suggest that the scientists engage more with users who are members of the public?
4. Do both groups of expert use a more informal and involved style of communication or are there any differences in their communicative styles?

## 3. Methodology

This section outlines the methodological steps that were undertaken to answer the above research questions. Based on the number of followers on Twitter, tweets by the top 25 scientists, (obtained by taking 25 top living scientists from Science magazine list published in 2014) and top 25 economists (from the IDEAS list which is updated daily; we have taken the list on 6 April 2017) were gathered into two data sets, from which only tweets containing mentions of people (including ‘@’) were extracted for the network. Retweets were disregarded, as were loops, that is, when a Twitter user mentioned themselves in a tweet. Our criterion for data collection was to select the last 3240 tweets^1^ from each person on the list (economists’ tweets ranged from 02/11/2009 to 07/03/2019, the scientists’ tweets from 09/12/2008 to 07/03/2019). Since there is a limit imposed by Twitter on a number of tweets that can be collected from individual profiles, we have roughly a similar amount of data per each scientist and economist. This, in turn, ensures that our analysis is not influenced by one or two individuals only. Table 1 shows the number of tweets by the top 25 scientists and top 25 economists, with tweets containing mentions (disregarding retweets and loops).

**Table 1.** Tweets (excl. retweets).

Data set	#tweets excl. RT	#words	#mentions	#users mentioned	#photos
Scientists (min, max, median)	53,314 (281, 3216, 2553.0)	1,078,188	40,652 (104, 2228, 902.0)	16,965 (67, 1834, 522.0)	4247 (8, 425, 56.0)
Economists (min, max, median)	42,736 (63, 3119, 1639.0)	1,252,649	26,871 (3, 2090, 440.0)	8,946 (3, 1119, 311.0)	4015 (0, 803, 36.0)

RT: retweet.

### Social network analysis

Our first question was: with whom do experts communicate? We performed a social network analysis (Wasserman and Faust, 1994) in order to find out. A social network is made of individuals (in network analysis also called nodes or vertices) and a set of connections between them (also called edges or links). In our case, the individuals are Twitter users, and they connect by mentioning each other. Two people can be connected through several mentions in each direction. From the collected tweets containing mentions, we created two evolving social networks, one for Economists, and one for Scientists. Each network contained a set of people (Twitter IDs) and directed connections between them were created using a following rule: if A mentioned B in a tweet in that week, a time-stamped, directed connection from A to B was created, therefore allowing for multiple connections between two individuals. One tweet could be used to create several connections if it contained several mentions. If it did not contain any mentions, the tweet was ignored. Next, we counted the number of connections that each person has, that is, how many times she or he mentions others and how many times she or he has been mentioned. The number of connections of A is obtained by summing up the number of mentions of A and the number of mentions by A. We also wanted to see how many different people A mentions, and we counted for each person how many different people they mention, that is, how ‘varied’ their communications are compared to the other people in the network. We call the two numbers (a number of total connections, and a number of different people that A is connected to) a ‘social signature’ of A.

At a group level, we are interested in identifying ‘communities’, groups of individuals/nodes who are more ‘tightly’ connected among themselves forming a ‘clique’ and more loosely connected to the rest of the network. For each network, we denoted 25 starting (top) Twitter IDs as *super-users*. As we did not collect the other people’s tweets, the other people in the network have only incoming connections (we can see only when they are mentioned, but not when they are mentioning others), and therefore, the number of connections going out is 0. In the next step, we extracted and analysed mentions that occur in tweets produced by both groups of experts independently to shed light on the identities of those who they mention and hence with whom they engage in conversation. The social network analysis and the analysis of mentioned Twitter users provide answers to research questions 1 and 3.^2^

Furthermore, we draw from a literature on affective dynamics – how a positive and negative mood spreads throughout a network (Alvarez et al., 2015; Golder and Macy, 2011; Kramer et al., 2014; Schweitzer and Garcia, 2010; Tadić et al., 2013; Vukadinović Greetham et al., 2015) to assess the difference in mood between the two groups. To do so, we processed all the tweets through Sentistrength (Thelwall et al., 2012), an open-source software that is developed specifically for Twitter and detects the sentiment of a given text, assigning to it both a positive and a negative score: 1 to 5 for positive and –1 to –5 for negative, thus fitting well with the positive and negative affect concepts.

### Semantic network analysis

To answer our second question – what kind of topics the two groups of experts communicate about – we turned to semantic network analysis. Improvements in machine-learning methods such as Google BERT (Devlin et al., 2019) together with curated large open corpora such as DBPedia and BabelNet have resulted in recent exciting developments in semantic web methods. Our study follows the approach of Jiang et al. (2018) and Calabrese et al. (2019); it is based on identifying the network of associations between concepts expressed in a text. We created a network of words, where two words are connected if they are in vicinity of each other in a tweet. First, the tweets were striped of stop words (e.g. ‘a’, ‘an’ ‘the’), different forms of the same word stem (e.g. ‘desire’ and desired’), lemmatised (combined to the dictionary form depending on the context) and tokenised. In this way, we obtained an ordered list of words for each tweet. We looked at the vicinities of words and created an undirected link between two words if they were first, second, or up to fifth neighbour of each other following Calabrese et al. (2019). We focused our analysis only on words that were most frequent in a given data set. For both data sets, we used a threshold 300 for a word’s frequency and obtained similar sized networks of 210 words and 14,000 links between them for economists and 196 words and 17,000 links for scientists. Once we constructed the network of words, we were able to identify the most important (or ‘central’) words that are connected to many others important words. We were also able to identify ‘clusters’ or ‘communities’ of words – the groups of words that are densely connected, that is, often tweeted together.

### Language analysis

In order to examine the communicative style adopted by the two groups of experts, two corpora of their Twitter posts were created and uploaded onto Sketch Engine – a linguistic software programme (Kilgarriff et al., 2004). This programme allows a number of comparisons to be made between two data sets in order to analyse differences (or similarities) in language use. One of the tools available on Sketch Engine is that of keywords and key terms. Keywords and key terms are words and two-word phrases that occur more often in one data set as compared to another, mostly large and representative reference corpus. Keywords and key terms retrieved in this way are seen as useful indicators of the thematic ‘aboutness’ of a corpus and highlight lexical items that are distinctive to the data sets under investigation. Comparing keywords and key terms in the corpora of tweets produced by both groups of experts can therefore reveal the lexico-grammatical specificity of language that they use and highlight features of their communicative styles.

We retrieved key words and key terms from both data sets using English Web 2015 as a reference corpus. This corpus is a compilation of texts sourced from the Internet and contains 15 billion words. It is a robust and representative corpus of the language found on English websites and hence a useful comparator, since the language that we study is an example of web language. Sketch Engine identifies keywords and key terms using a normalised frequency ratio ‘word W is N times as frequent in corpus X versus corpus Y’ with a simple math parameter added to account for the zero problem in divisions (Kilgarriff, 2005). Using the tool, we retrieved the top 100 keywords and key terms from both data sets. Subsequently, the retrieved keywords and key terms were manually classified into the markers of formality or informality. Formality features include domain-specific names, terminology and abbreviations, while features of informality are discourse markers and informal forms of address, personal pronouns and emotive/evaluative lexis. While domain-specific lexis requires prior knowledge of the field and adds to the exclusivity of ‘talk’, discourse markers (*yeah, oh, so*), forms of address (*hello, hi*), politeness markers (*thanks, sorry*), the use of emotive and evaluative lexis and personal pronouns are features of involvement that can foster engagement. The grouping of the key words and terms into the domains was conducted first independently by the three researchers; disagreements and ambiguous meanings were resolved by checking the meanings of the key words and key terms in context, that is, how they were used by the scientists and economists. For example, the word ‘cool’ was placed into the category emotive/evaluative lexis because in most of the instances of its use it was an evaluator and not a descriptor of temperature.

## 4. Results

### Who do scientists and economists communicate with?

We report the average number of connections (mentions) of economists’ and scientists’ social networks in Table 2. Top tweeting scientists interact with more people than top economists (higher number of individuals in a network) and have more communications in total (higher number of connections). On the other hand, more mentions were observed for a top ‘mentioning’ economist than for a top ‘scientist’. The two sets of super-users had similar ratios of the followers/followees (see Supplemental material, Figure 3) thus, their importance on Twitter was perceived as similar across two groups.

**Table 2.** Basic network statistics.

Network	No. of individuals	No. of connections	Avg. no. of connections per person	Max number of connections by one person
Economists	8971	26,871	5.99	4663
Scientists	16,990	40,652	4.79	3562

**Figure 1.**
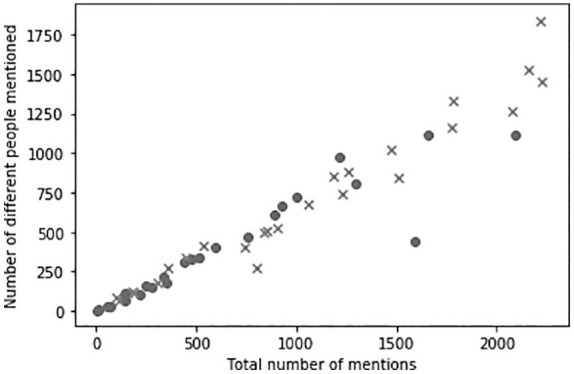
On *x* axis, the total number of mentions, on *y* axis, the number of different individuals that were mentioned (economists are represented with dots and scientists with crosses).

Do the experts talk to (mention) the same people repetitively, or do they have a large social circle? In order to answer this question, we calculated social signatures. We plotted the social signatures for both groups (see Figure 1). While both groups have similar plot, at the top of ranks economists differ from scientists in that they have more ‘conversations’ with the same users, that is, mention more often the same people.

**Figure 2.**
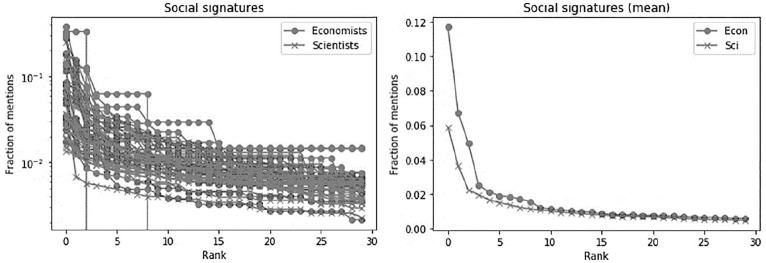
Social signatures.

As this can be an artefact of choosing one discipline (Economics) and more different disciplines (Science), we also explore the individual social signatures in both groups in more details, using the methods described by Saramäki et al. (2014). Social signatures are constructed for each super-user by counting the number of mentions to other people. We then ranked the mentioned people based on this number and calculated the fraction of mentions to the mentioned person of each rank. This can be seen on the right panel of Figure 2. On average, across their whole cohort, a fraction of mentions of the same most mentioned people is higher for economists. This suggests that the top economists are more likely to engage with the same people and stay in their own ‘conversation bubbles’.

#### Mentions

Mentions are a useful way to establish the points of reference of users, and of course we expect to see a difference between the two groups regarding top mentioned accounts. One interesting aspect of this difference, however, is that scientists mention more videos than economists: while for Scientists YouTube is in the top 7, for Economists it does not make it to the top 20. Snapshots of two mentions networks are given in the Supplemental material in Figures 1 and 2. While the economists’ network is much more spread out and tree-like (super-users’ frequently mentioned individuals do not overlap much), the scientists’ network is more compact, with more connections across different neighbourhoods. The latter means that the diameter of the scientists’ network is actually smaller, that is, one can reach from any person in the network to another person in the shorter number of hops. On the other hand, we would expect the opposite to be true, as the scientists are from different disciplines.

#### Sentiment

Sentistrength scores on tweets were used to create an average positive and negative sentiment score for each super-user by calculating the means of these two scores. A *t*-test for means of two independent samples from descriptive statistics (mean and standard deviation) shows that Economists’ positive sentiment mean of 1.3513 was significantly lower than Scientists’ mean of 1.5516 (test statistic = –4.6966, *p* = 2.2470e–05). Economists’ mean for negative sentiment score was slightly higher, but not significantly (–1.4080 vs –1.4208, test statistic = 0.2528, *p* = 0.8015). It was noted by Charlton et al. (2016) that on average top broadcasters (people with largest outreach) send positive sentiment messages more often, and negative sentiment messages less often. In addition, when they use positive sentiment, it tends to be stronger, so this could be a potential area of concern for economists.

#### Semantic network analysis

The economists’ semantic network was split into four communities – groups of words densely connected between themselves and loosely connected to the rest of the networks: the first community (17% of the network) contained Spanish words; the second (13%) contained terms related to ‘book’, ‘chapters’, ‘file’, ‘downloads’ and financial and behavioural economics; the third was relatively broad (63%) with a lot of general terms (e.g. ‘future’, goods’, ‘foods’, ‘government’) and the fourth (7%) contained ‘capital’, ‘class’, ‘Marx’, ‘reason’ and words related to video and lectures (see the Supplemental material, Table 1).

The scientists’ semantic network was split into five communities: the first community (8% of the network) contained space-related words (‘space’, ‘moon’, ‘planet’, ‘star’); the small second (1.5%) contained ‘question’, ‘answer’ and ‘ask’; the third community (21%) had general terms about outreach (e.g. ‘podcast’, ‘school’, ‘talk’, ‘live’, etc); the fourth (31%) contained terms related to medicine and physics; and the fifth contained general terms (38%) such as ‘woman’, ‘world’, ‘president’, ‘country’ and so on.

This shows among other things that the economists refer to traditional media of knowledge dissemination such as books and chapters and distribute them for download, while the scientists tend to refer to multimodal media of communication. This is confirmed by the keyword analysis below.

### How do scientists and economists communicate?

The analysis of keywords and key terms reveals that both groups of experts use frequently domain-specific names and terminology which points to a more formal and exclusive style of communication (tables with the top 100 keywords can be seen in the Supplemental material). Yet, when examining and categorising the words into the specified categories, it was observed that the scientists offset the formal style using features associated with informality and involvement and thus rendering their language more accessible. Scientists’ tweets appear to use frequently evaluative and positive language often indicating excitement and enthusiasm; words such as *amazing, fantastic, awesome, fascinating* occur regularly in scientists’ tweets. This tendency is absent from economists’ keywords suggesting that economists might abstain from using positive evaluation. The only positive evaluator found in the top 100 keywords in the economists’ tweets was *interesting*, which indicates a moderate evaluation. Examples of tweets below are indicative of the differences in styles:

#### Scientists’ tweets

1. The fascinating crosstalk between stem and immune cells: it ‘takes 2 to tango’.
2. Wow! Very dramatic. The crescent Moon was lovely last night.
3. Thank you:-) Perspective is always valuable

#### Economists’ tweets

4. Interesting World Bank paper on the challenge of skewed distribution of global talent.
5. This is a chart of Q1 tax revenues (personal + corp + payroll) by fiscal year. Not adjusted for inflation.
6. Even as ordinary labour and capital get lower returns, the owners of a third factor, G, are getting superstar returns.

Also, scientists’ tweets show several other informal features among the top keywords such as the use of politeness markers and personal pronouns, which point to a more interactive style of their Twitter communication. In contrast to economists, who show fewer of these devices, scientists seem to try and engage in conversations and acknowledge others as opposed to just imparting information. The category ‘other’ too reveals some interesting trends regarding the communicative style of both expert groups. Among the top 100 keywords in scientists’ emails, we find many items that point to different digital (e.g. *podcast, Facebook, YouTube, blog, app*) and public media (*BBC*). What the top scientists often do is to provide links to contents that impart information in multimodal ways, for example, through videos and audios. While they too share contents available in a more traditional medium (e.g. *book, article*), they also try to encourage audiences to watch and listen (*watch, talk, conversation, interview, lecture*). The economists seem to be more preoccupied with sharing information from traditional media (e.g. *pdf, column, review, book, article, overview*). In short, they mostly want their audiences to read ‘stuff’ from written sources including newspapers (*FT, NYT*). In terms of similarities, there are two themes on which both groups seem to converge: the engagement with political matters and AI (see the Supplemental material, Table 2).

The differences are confirmed when looking at the top 100 key terms (the top 100 key terms can be seen in the Supplemental material, Table 3). While both groups of experts use domain-specific names and terminology, economists’ tweets employ more terminology and also more proper names, mostly names of important economists who, however, might not be well known to the general public. Conversely, scientists intersperse factual tweets with tweets that are much more personal and engaging in style. They evaluate (most positively) contributions from other Twitter users and promote certain contents. They also engage in small politeness talk, as the instances of polite key terms indicate (e.g. *thank you, good luck* etc). Also, the key terms highlight the different ways of sharing information. While many of the key terms used by scientists point to a variety of media and channels (e.g. *new book, new paper, live stream, ted talk, quirkology video, show tonight*), economists make references almost exclusively to books. This suggests that reading (possibly of specialised contents) is the key literacy skill that is required to process the contents shared by economists. While there is nothing wrong with sharing contents from books, most of the contents that economists distribute refer to specialised literature which might not be easily accessible to people outside this group of experts.

#### Pronoun usage

Personal pronouns are the paramount linguistic devices of indexicality. Previous research on the use of pronouns in public discourse has shown that they act as important tools of signalling and maintaining individual or group membership (Cramer, 2010; Fetzer and Bull, 2008; Jaworska and Sogomonian, 2019; Tyrkkö, 2016). Greater use of personal pronouns, especially first- and second-person pronouns, is considered indicative of an involved style, whereas lesser use of these devices points to a nominal and impersonal style (Biber and Conrad, 2009).

We therefore investigated the use of first- and second-person pronouns in both data sets to see whether there are any differences in the use of this feature indicating a more personal style of one group as compared to another.

From the figures in Table 3, we can observe statistically significant differences in the use of first- and second-person pronouns across both groups of experts. While scientists use more first-person singular pronouns, which indicates a more self-centred style than that of economists, scientists also use a far greater proportion of second-person pronouns. Since second-person pronouns address interlocutors directly, this points to greater addressivity - i.e. directed to a listener - and involvement in scientists’ tweets (Bakhtin, 1986). Also, scientists use far more first-person plural pronouns demonstrating more of a group-centred style than economists. Pronouns such as *we* and *our* are often employed in public discourse including Twitter communications whether there is a need or a goal to construct and reinforce collectivity and shared understanding (Jaworska and Sogomonian, 2019). The greater use of *we, our* and *ours* implies a much more inclusive style exhibited by the scientists that is likely to engage people outside that group. It could also create the sense of togetherness and shared experience, thus indicating a more involved stance.

**Table 3.** The use of first- and second-person pronouns.

Pronoun	Scientists raw Freq.	Scientists norm. Freq.^a^	Economists raw Freq.	Economists norm. Freq.	Log Likeli-hood^b^	BIC	*pd*
First-person singular (I, me, my, mine)	15,091	14.0	13,798	11.0	414.12	399.45	****
Second person (you, your, yours)	12,086	11.2	5956	4.8	3145.52	3130.86	****
First-person plural (we, us^c^ our, ours)	8711	8.1	5264	4.2	1454.55	1439.88	****

BIC: Bayes Factor BIC to test the effect sizes.

a Since the sizes of the two data sets were unequal in terms of the number of words, raw frequencies were normalised per 1000 words.

b Log likelihood metric relies on the normalisation procedure based on total corpus size, hence is well suited to deal with large quantities of non-parametric data. It is a good measure for testing the significance of differences between token counts of specific linguistic features across corpora (Dunning, 1993).

c Instances in which ‘us’ meant ‘USA’ were removed.

d The following notation has been used to indicate the level of statistical significance: * *p*<0.05; ** *p*<0.01; *** *p*<0.001; **** *p*<0.0001.

The data from the above analysis implies that the communicative style of economists, tends to be more distant and less personal and inclusive. Their language on Twitter exhibits a number of features that are representative of a more formal communicative style, while scientists additionally employ some highly informal language in their tweets. Scientists use a notably larger number of first-person plural pronouns such as *we* and *our* demonstrating a higher degree of affiliations and togetherness.

## 4. Conclusion: Lessons to be learnt

Social media are a new medium through which public influence is exercised, and it is important that experts, especially those who influence public policy such as scientists and economists, are aware of what makes their messages more effective in reaching the public, especially at times when the fight for people’s attention is so clearly intensifying (Wu, 2017), and the level of public understanding of what experts do is rather low.

In our Twitter study, we compared economists to scientists, who are arguably ahead in the use of social media sites and made use of language, network, sentiment and semantic analysis to understand patterns of their communications on Twitter. Despite the fact that both groups in our study belong to the most followed scientists and economists, they seem to have developed different communicative practices. Our analysis shows that despite similar tendencies to communicate with a wide range of people, economists tweet less, mention less and mention fewer users and communicate with lower sentiment. Our language analysis of *differences in communicative style* finds that economists use a more formal and exclusive style of communication than scientists. *Differences in pronoun use* demonstrate that highly-inclusive pronouns such as *we* and *our* are used up to twice as often by scientists when compared to economists indicating more engagement between the tweeting scientists and their audiences. While we do not assume that scientists are always more engaging, the most followed ones seem to display a more involved style of communication, which could explain their wider reach and accessibility. Economists, on the other hand, seem to be more exclusive. If there is a lesson to be learnt, they need to focus their communication on talking more with people (rather than at them), show they care about the issues at hand (and remember people do too) and worry more about being understood by non-specialists when wishing to engage with the public. One suggestion stemming from this comparison is that parallel to sharing contents from specialised literature, experts across disciplines could engage the audiences in a more multimodal presentation and explanation of subject matters, which can make them more accessible to non-experts. While our findings are limited to a data set including only the top 25 users with the largest following, and their latest 3420 tweets, they chime with the results of the climate survey recently implemented by the American Economic Association. It would of course be interesting to see if all the tweets per person, or a random sampling of users with different numbers of followers would lead to similar conclusions. More insights could also be learnt by complementing this analysis with retweets and focusing on the dynamics of sharing simultaneously with the dynamics of conversations by mentions with a larger data set and more history.

## Supplemental Material

PUS_Supplemental_Material_19-0130.R2 – Supplemental material for Expert communication on Twitter: Comparing economists and scientists’ social networks, topics and communicative styles Click here for additional data file. Supplemental material, PUS_Supplemental_Material_19-0130.R2 for Expert communication on Twitter: Comparing economists and scientists’ social networks, topics and communicative styles by Marina Della Guista, Sylvia Jaworska and Danica Vukadinović Greetham in Public Understanding of Science

## Notes

1. This limit is imposed by Twitter API.

2. For readers who want to know more about the social network analysis, the seminal reference is Wasserman and Faust (1994). For general network analysis, Brandes and Erlebach’s study (2005) is a practical and accessible textbook with breadth of methods described in detail but it does require some mathematical background.
