# Supplementary material for: Expert communication on Twitter: Comparing economists’ and scientists’ social networks, topics and communicative styles
Source: Public Underst Sci. 2020 Sep 15;30(1):75–90. doi: 10.1177/0963662520957252 (PMC7802044; doi:10.1177/0963662520957252)
Supplement: PUS_Supplemental_Material_19-0130.R2 – Supplemental material for Expert communication on Twitter: Comparing economists and scientists’ social networks, topics and communicative styles [file PUS_Supplemental_Material_19-0130.R2.pdf]

Supplemental Material

Expert communication on Twitter: comparing economists and scientists' social networks, topics and communicative styles

Marina Della Guista
Sylvia Jaworska
Danica Vukadinović Greetham

1. Network and Sentiment Analysis

1.1. Network Analysis

At individual level, centrality measures the relative importance of an individual (a node) and determines its involvement in a network. We calculate three types of centrality rankings: in-degree, out-degree, degree; hub-authorities centrality; eigenvector and Page-rank centrality.

The simplest centrality measures are just counting the connections. The in-degree of A simply counts how many times was other users have mentioned a user A mentioned, while the out-degree of A counts how many times other users were mentioned by a user A mentioned others. The degree of A is the sum of the in-degree of A and out-degree of A. However, in real social networks, sometimes the most important person might not be the most connected person. It might also be important to what type of person they are connected to. A popular centrality measure inspired by the analysis of the World Wide Web and based on HITS algorithm (Kleinberg, 1999), is hub and authority centrality measure that combines in and out-degrees. It assigns a higher authority measure to an individual that, which is mentioned by many different people that mention others a lot (thus having a large out-degrees). In lay terms, a hub is an individual/node that points out to many others, and an authority is an individual that is pointed by many different hubs.

Another commonly used centrality measure is Eigenvector centrality. The main idea of this centrality measure is that the centrality of a node depends not only on the number, but also on the centrality of nodes connected to it. Intuitively, your importance grows if you are connected to important people. Similar to eigenvector centrality, Page-rank (Page et al., 1999), "one of the main ingredients of the search engine Google" (Brandes and Erlebach, 2005) takes into account the centrality of each node connections, while also dealing with some issues of eigenvector centrality.

We included only the users that were mentioned at least 20 times across the whole period. The size of nodes (individuals) corresponds to their Pagerank index (a measure of centrality, the bigger the node, the larger the Pagerank[1] index). The darker the colour the nodes denotes higher normalised Authority[2] index. The thickness of links is proportional to the number of mentions (see Figures 1.1.1. and 1.1.2).

---

[1] PageRank is a centrality index, one of the main concepts of Google search (Page et al., 1999). The main idea of this centrality measure is that the centrality of a node depends not only on the number, but also on the centrality of nodes connected to it, forming a feedback on the network.

[2] Based on HITS algorithm (Kleinberg, 1999), authority is a centrality measure that assigns a higher value to a node (individual) that is mentioned by many different nodes (individuals) that mention others a lot so having outgoing high

At group level, we can identify ‘communities’, groups of individuals/nodes who are more densely connected among themselves and more loosely connected to the rest of the network. For readers who want to know more about the social network analysis the seminal reference a is (Wasserman and Faust,1994). For general network analysis (Brandes and Erlebach, 2005) is a practical and accessible textbook with breadth of methods described in detail, but it does require some mathematical background.

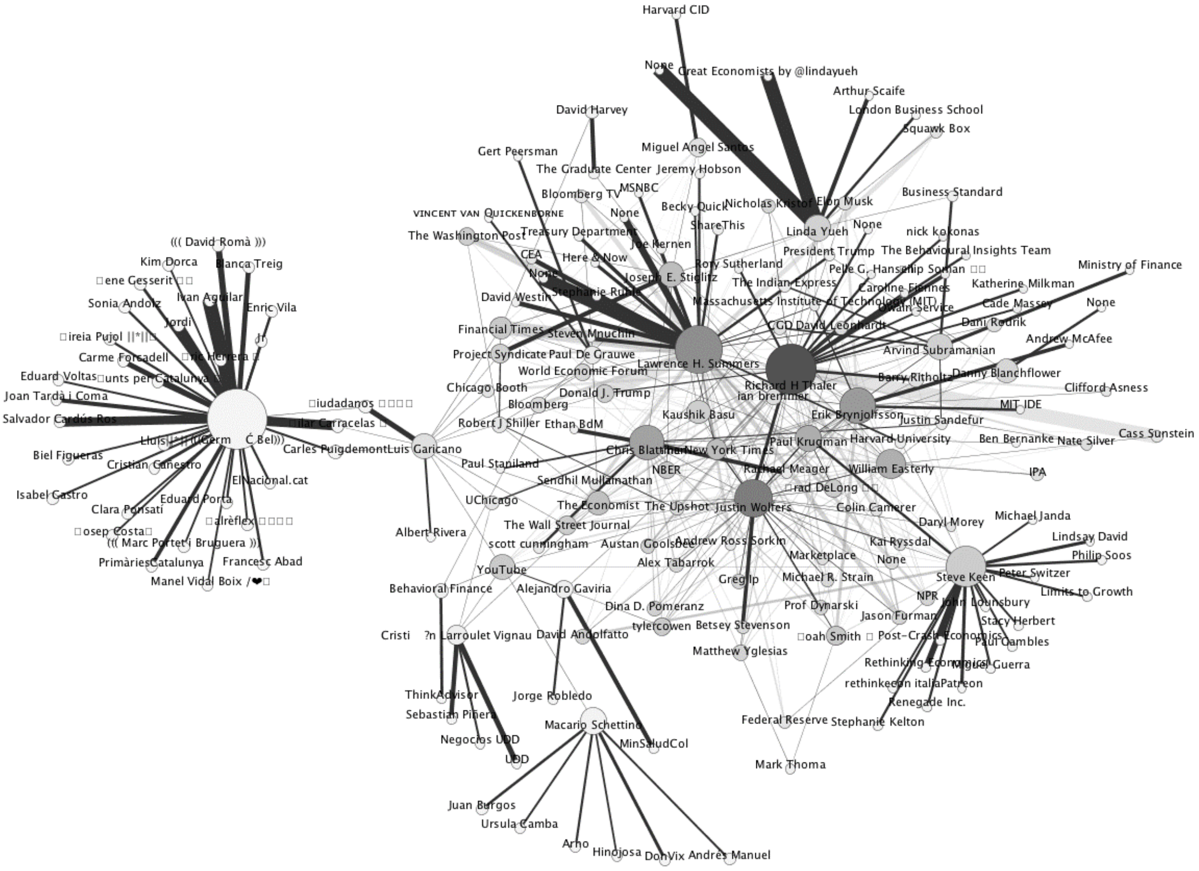

Figure 1: Economists - mention network with accounts that were mentioned more than 20 times.

centrality, or in lay terms, a hub is a node that points out to many other nodes, and an authority is a node that is pointed by many different hubs.

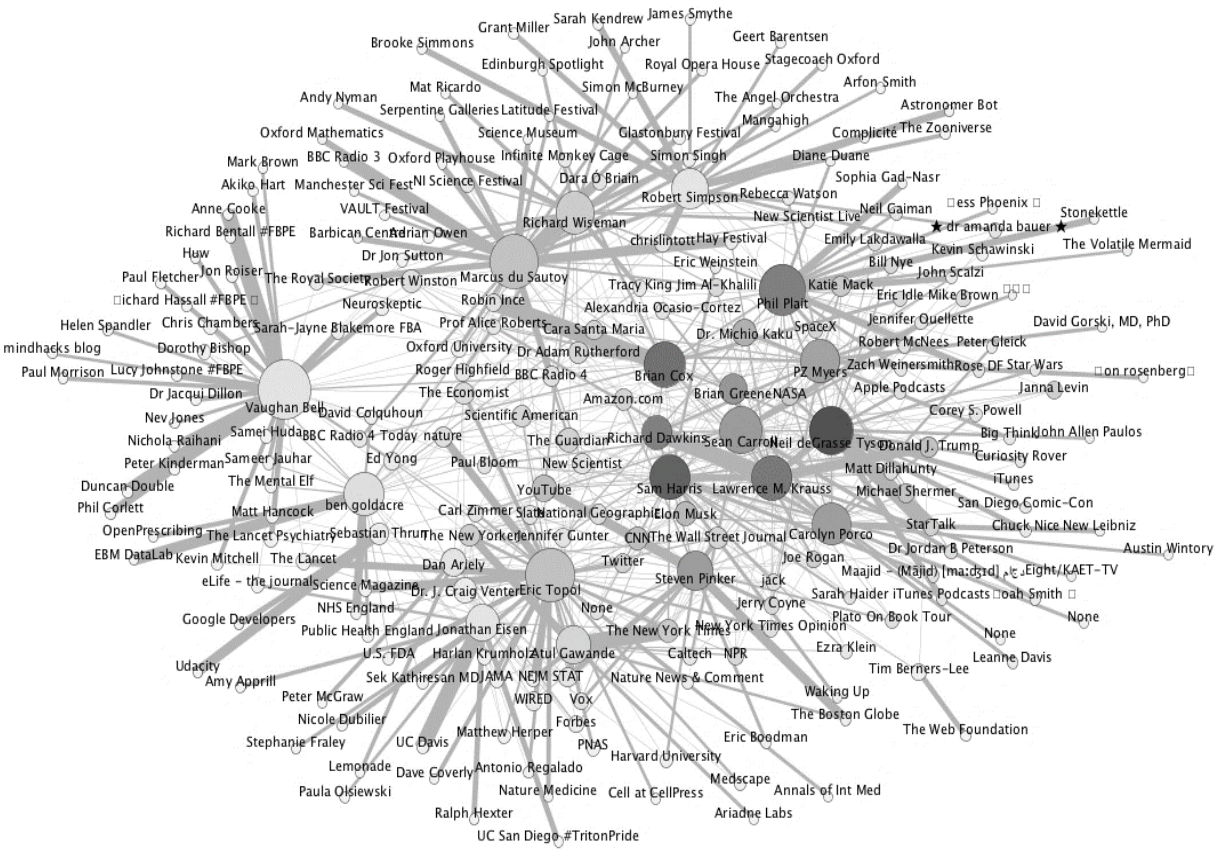

Figure 2: Scientists - mention network with accounts that were mentioned more than 20 times

1.2. Sentiment analysis in social networks

In social networks, sentiment or mood is sometimes described using as proxies positive and negative affect, ephemeral constructs that are easy to measure (Watson et al., 1988) and relate to the usually short-lasting emotions or feelings that we experience and display. In the social psychology literature, they are used to connect mood and well-being (De Neve and Cooper, 1998; Tellegen, 1985).

The two affects follow different dynamics in social groups (Vukadinović Greetham et al., 2011) and are not complementary, so one can score at the same time high on both positive and negative affect. While in small group self-reported PANAS questionnaires (Watson et al., 1988) can be used to quantify affects in a particular time, in Twitter corresponding measures are usually unobtrusively obtained using averages of sentiment expressed in tweets per individual. It should be noted that due to its nature (e.g. shortness of the text, informality, use of emojis etc), Twitter presents sentiment classifiers with several challenges. Our chosen, open-source tool to infer Tweets sentiment is Sentistrength. As noted in Ferrara and Yang (2015), Sentistrength is optimised to annotate short informal texts, that contain abbreviations, slang, sarcasm etc., and it includes linguistic rules for negations, amplifications, booster words, emojies, spelling corrections, etc. Cross-validation of Sentistrength with other two sentiment classifiers (including commercial one) on large Twitter datasets presented in Charlton et al. (2016) has affirmed our choice.

1.3 Similarities of the two networks: the ratio of followers and friends

How important are the super-users in the two networks? Is there a difference in their perceived ‘authority’ on Twitter?

The suggestion by Westerman et al. (2012) that the number of friends should match in magnitude the number of followers in order to be perceived as an expert do not apply to Twitter users with huge following, as it is physically not possible for humans to meaningfully follow huge numbers of accounts.

For each super-user we looked at the ratio of followers (Twitter users that follow that super-user) and ‘friends’ (Twitter-users that the super-user follows). As the super-users in our sets can be seen as ‘celebrities’ (for Economists and Scientists respectively, the maximum number of friends is ~85k and ~7k and the maximum number of followers is ~4.5M and 13M) in accordance with Saito and Masuda (2014) they can be seen as ‘Type 1’ users – users with large following, a small number of friends, and large centrality index.

Looking at the scatter plot of the ratio of followers/friends (see Figure 3, log y scale is used because of range) shows similar behavior for both sets, thus the two sets are comparable regarding their importance on Twitter.

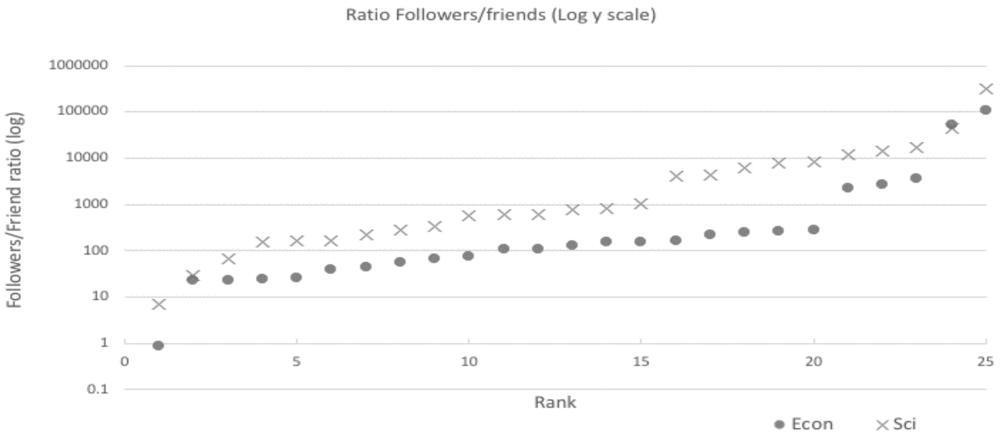

Figure 3: Followers/Friends ratio for super-users in both sets (economists are represented with the dots and scientists with crosses).

2. Results of the semantic network analysis

Table 1: The 20 most central words

**Eigenvector Centrality**	**Economists**	**Eigenvector Centrality**	**Scientists**
**1.00**	new	1.0000	people

**0.99**	economic	1.0000	time
**0.99**	great	1.0000	good
**0.98**	world	1.0000	need
**0.98**	read	1.0000	right
**0.98**	trump	0.9959	life
**0.98**	today	0.9958	day
**0.98**	book	0.9958	want
**0.98**	economics	0.9957	like
**0.97**	change	0.9950	world
**0.97**	economist	0.9906	get
**0.97**	time	0.9905	come
**0.97**	paper	0.9903	way
**0.97**	need	0.9903	yes
**0.97**	real	0.9861	book
**0.97**	years	0.9851	love
**0.96**	idea	0.9814	help
**0.96**	like	0.9805	big
**0.96**	think	0.9785	read
**0.96**	economy	0.9779	story

3. Results of the corpus analysis using Sketch Engine

Table 2: The top 100 keywords

	**Scientists**	**Economists**
domain specific names, titles, abbreviations and terminology	universe, science, brain, maths, moon, genome, earth, cancer, scientist, phylogenomics, quantum, planet, physics, NASA, climate, space, medicine, phylogenomic, genomic, AI, Mars, Pinker, mental, microbiome, Cox, Kaku, Einstein, galaxy, evolution, cosmic, Dr	Ricciardi, behaviour, investor, Harvey, Victor, economists, GDP, nudge, investing, financial, finance, capitalism, fiscal, debt, poverty, AI, capital, macro, Euro, infrastructure, trade, Marx, Obamacare, tax, policy, govt, biases, Summers, Eurozone, recession, Krugman, theory, Prof., econ, investor, economic, wage, money, deficit, money
discourse markers and forms of address	yeah, hey, yup	hi
politeness markers	thank, congrats, sorry	thank
personal pronouns	my, you, we	-
emotive/evaluative lexis	wow, amazing, interesting, fun, excellent, fascinating, glad, awesome, nice, happy, fantastic, ok, lovely, great, favourite, wonderful, cool, brilliant	interesting

other	tweet, Twitter, tonight, today, video, interview, blog, podcast, online, email, Obama, google, article, read, piece, book, photo, Brexit, lecture, Islam, talk, news, tomorrow, datum, vote, human, post, GOP, Muslim, Trump, app, story, youtube, BBC, Internet, review, pm, watch, conversion, religion, atheists, kid, Facebook, Harvard	Trump, GOP, Brexit, EU, pdf, blog, downloadable, Venezuela, global, tweet, interview, Clinton, China, Twitter, Chile, book, India, podcast, Obama, today, UK, read, Donald, pm, FT, file, links, review, expert, debate, Potus, post, Columbia, overview, column, vote, news, USA, retirement, president, NYT, voter, article, Nobel, robot, links, chapter, paper, thread

Table 3: The top 100 key terms

	**Scientists**	**Economists**
domain specific names, titles, abbreviations and terminology	mental health, black hole, deep learning, climate change, dark matter, human genome, health care, clinical trial, Timberners Lee, solar system, Larry Levitt, artificial intelligence, life expectancy, polygenic risk, prime number, wave function, Tom Watson, citizen science, public health, machine learning, natural selection, general relativity, cognitive science, open access, solar eclipse, dark energy, tax bill, bryan johnson, open science, peer review, big bang, trial reporting, neural network, brain injury, full moon, space station, cancer immunotherapy	Ricchardi's book, Bill Easterly, behavioral finance, poverty action, Ricchardi's article, Marx's capital, trade war, tax cut, Ricchardi's (TV) appearance, secular stagnation, Ricardo Hausman, Austan Goolsbee, adviser behaviour, dany bahar, wage growth, tax bill, tax reform, financial therapy, (bad) client behaviour, corporate tax, foreign aid, Kennedy school, global poverty, trade policy, next recession, Katy Milkman, monetary policy, critical retirement, mental accounting, trade deficit, political economy, fiscal policy, financial literacy, game theory, Leah Boustan, investing psychology, economic policy, republican tax, basic income, Gabriel Zucman, productivity growth, trade deal, inflation target, estate tax, wealth management, economic history, job market, chief economist, economic development, job growth, comparative advantage, economic crisis, income inequality, middle income, economic theory, full employment, machine learning, poverty rate, behavioral investor, Judy Chevalier, exchange rate, world economy, national security, child mortality, infrastructure investment, Prof Karthik, expert economist, political science, Ricchardi's blog, carbon tax, Billy blog, international trade
politeness markers	thank you, good luck, happy birthday, huge congrats, (have a) great day	-
emotive/evaluative terms	very kind, good point, good piece, good news, interesting piece, great piece, great work, excellent piece, great conversation, good question, standing ovation, great news, great interview, interesting study, great stuff, amazing work, big news, good thing, bad science, important study, excellent review	great piece, dangerous mix, timely read, interesting thread, great news

other	new book, last night, next week, free speech, bmj latest, book tour, new paper, unreported trial, new study, blog post, fake news, astro nerd, national radio, ted talk, recent conversation, direct link, nope nope, radio show, new series, sexual harassment, tomorrow night, tomorrow morning, free will<br>audio player, next time, gun violence, news story, brief explanation, public lecture, hayfestival esp, live stream<br>next book, quirkology video, new play, great audience, crescent moon, show tonight	book chapter, book review, positive book, new book, introduction chapter, advisory board, blog posting, book talk, fake news, finance book, current book, (current) book chapter, podcast series, facebook page, brief history, grad school, interview with, 3d printing, discussion with, lecture series, working paper, tv program, 21st century

References

Kleinberg JM (1999) Authoritative sources in a hyperlinked environment. Journal of the ACM 46: 604–632.

Page L, Brin S, Motwani R and Winograd T (1999) The pagerank citation ranking: bringing order to the web. Technical Report. Stanford InfoLab. Available at: http://ilpubs.stanford.edu:8090/422/ (accessed 7 July 2019).

Brandes U und Erlebach T (Eds.) (2005) Network Analysis - Methodological Foundations, LNCS Tutorial 3418, Springer Verlag.

Wasserman S and Faust K (1994). Social Network Analysis: Methods and Applications (Structural Analysis in the Social Sciences). Cambridge: Cambridge University Press.

Watson D, Clark LA and Tellegen A (1988) Development and validation of brief measures of positive and negative affect: The PANAS scale. Journal of Personality and Social Psychology 54, 1063–1070.

De Neve KM and Cooper H (1998) The happy personality: a meta-analysis of 137 personality traits and subjective well-being. Psychological Bulletin 124: 197–229.

Tellegen A (1985) Structures of mood and personality and their relevance to assessing anxiety, with an emphasis on self-report, Hilsdale, pp. 681–706.

Vukadinović Greetham D, Hurling R, Osborne G and Linley A (2011) Social network and positive and negative affect. Procedia-Social and Behavioural Sciences, Dynamics of Social Network Analysis 22: 4–13.

Ferrara E and Yang Z (2015) Measuring emotional contagion in social media. PLOS ONE 10(11): e0142390, https://doi.org/10.1371/journal.pone.0142390

Charlton N, Singleton C and Vukadinović Greetham D (2016) In the mood: the dynamics of collective sentiments on Twitter. Royal Society Open Science 3(6): 160–162.

Westerman D, Spence PR and Van Der Heide B (2012) A social network as information: The effect of system generated reports of connectedness on credibility on Twitter. Computers in Human Behavior 28(1): 199–206.

Saito K and Masuda N (2014) Two types of well followed users in the followership networks of twitter. PLOS ONE 9(1): e84265.https://doi.org/10.1371/journal.pone.0084265
